# Transiently Evoked Otoacoustic Emissions in Children with Otitis Media with Effusion

**DOI:** 10.1155/2012/269203

**Published:** 2011-12-01

**Authors:** Dimitris G. Balatsouras, George Koukoutsis, Panayotis Ganelis, George S. Korres, Andreas Aspris, Antonis Kaberos

**Affiliations:** ^1^ENT Department, Tzanion General Hospital of Pireaus, Afentouli 1 and Zanni, 18536 Pireaus, Greece; ^2^ENT Department, Atticon University Hospital of Athens, 1 Rimini Street, Haidari, 12462 Athens, Greece; ^3^ENT Department, Nicosia General Hospital, Nechrou Avenue, Southeastern Nicosia, 1102 Cyprus, Greece

## Abstract

*Introduction*. Otitis media with effusion is a common pediatric disease whose diagnosis is based on pneumatic otoscopy, pure-tone audiometry, and tympanometry. The aim of this study was to evaluate transiently evoked otoacoustic emissions in the diagnosis of otitis media with effusion as compared to tympanometry. *Patients and Methods*. 38 children with bilateral otitis media with effusion were studied. 40 normal children of similar age and sex were used as controls. All subjects underwent pneumatic otoscopy, standard pure-tone audiometry, tympanometry, and transiently evoked otoacoustic emissions. *Results*. In the group of children with bilateral otitis media, transiently evoked otoacoustic emissions were absent in 51 ears (67%). In the remaining 25 ears (33%) the mean emission amplitude was reduced, as compared to the mean value of the control group. *Conclusions*. Transiently evoked otoacoustic emissions should be included in the diagnostic workup of otitis media with effusion because it is a fast, reliable, and objective test. Transiently evoked otoacoustic emissions should always be used in conjunction with tympanometry, because a more meaningful interpretation of transiently evoked otoacoustic emissions measures is possible.

## 1. Introduction

Otitis media with effusion is a common pediatric disease and is considered the most common cause of hearing impairment among children [1]. Diagnosis is mainly based on pneumatic otoscopy, pure-tone audiometry, and tympanometry. Tympanometry is an objective technique that can detect abnormal middle-ear function consistent with the presence of fluid in the middle-ear cavity [2].

Transiently evoked otoacoustic emissions (TEOAEs) are a diagnostic method widely used during the past decade to study cochlear function, in a noninvasive and objective manner. Usually, TEOAEs are present in people who have normal cochlear function and a healthy middle ear [3]. Although extensive experience from the use of TEOAEs in widespread neonatal hearing screening has been gained [4], little data has been gathered from the population of preschool and school-aged children. Because otoacoustic emissions are transmitted from the cochlea to the external ear canal via the middle ear, the transmission properties of the middle ear directly influence their characteristics. In general, middle-ear effusion reduces measured emission amplitudes and sometimes eliminates the response entirely [5]. The aim of this study was to evaluate TEOAEs in the diagnosis of otitis media with effusion in comparison with tympanometry.

## 2. Material and Methods

A group of 38 children with bilateral otitis media with effusion was studied. Twenty-one of them were male and 17 female, ranging in age from 4 to 15 years, with a mean age of 8.3 years. Forty normal children of similar age and sex were used as controls. Both patients and controls underwent clinical otologic and audiological evaluation including medical history, pneumatic otoscopy, tympanometry, and standard pure-tone audiometry. The diagnosis of otitis media with effusion was established when findings in at least three of them were positive. This was used as the gold standard for the comparison of the diagnostic accuracy of TEOAEs, tympanometry, or their combination.

Conventional pure-tone audiometry was conducted in a standard sound proof booth, using a two-channel Amplaid 455 audiometer and earphones. Standard audiometric procedures were applied and the pure-tone thresholds of each ear at frequencies of 0.25, 0.5, 1, 2, 3, 4, and 8 kHz were measured. Subjects were considered to have a hearing loss if any threshold between 250 and 8000 Hz exceeded 20 dB HL. When air conduction thresholds were out of normal hearing range, bone conduction thresholds were obtained.

Standard single-frequency tympanometry was performed with an Amplaid 770 clinical admittance meter, using a single frequency 85 dB SPL (sound pressure level) tone set at 226 Hz. The range of ear canal pressure was +400 to −600 daPa. The American Speech-Language-Hearing Association guidelines were used to determine if a tympanogram was considered abnormal [6]: (1) static admittance less than 0.3 mmho; (2) an equivalent ear canal volume greater than 1.0 cm^3^ when accompanied by a flat tympanogram; (3) tympanometric width greater than 200 daPa.

TEOAEs were further performed in all patients and controls, using a DP Echoport ILO 292 Otodynamics analyzer connected to a portable personal computer. The acoustical stimulation, the data recording, and the data analysis were produced automatically with the aid of this system. Testing was performed in a sound-treated room using a standard ILO adult probe with disposable tips. Meatus response monitoring was used to check fitting conditions of the probe. The noise rejection level at the probe tip was set to 47 dB. Stimuli were half-sinusoidal clicks of 100 *μ*sec duration. The nonlinear method of recording was used, allowing the phase-locked cochlear component of the response to be measured. The recording bandwidth was set between 0.75 to 5 kHz, stimulus intensity was approximately 80 dB, and repetition rate was 50 stimuli/sec. The numbers of responses accepted and rejected by artefact rejection were displayed and updated during averaging. The test was concluded after 260 total sweeps had been recorded. Details of this procedure are reported elsewhere [7]. The “pass” criteria were signal-to-noise ratio ≥6 dB, in four of five 1/2 octave frequency bands at 1, 1.5, 2, 3, and 4 kHz [8].

## 3. Results

Thirty-six patients were successfully tested with standard pure-tone audiometry. The remaining two were younger subjects who failed to respond adequately. However, pneumatic otoscopy, tympanometry, and TEOAEs were performed to both of them successfully, and the diagnosis of otitis media with effusion was established. Audiometry was successfully completed in all the subjects of the control group. Mean pure-tone thresholds exceeded 20 dB HL for the lower and middle frequencies in the group of children with otitis media with effusion, whereas mean values lower than 20 dB HL were found in the control group across all the examined frequencies (Table 1). In 72 ears (94.7%) of the group of patients the tympanograms were abnormal and in the remaining 4 (5.3%) they were normal. In 70 ears (87.5%) of the control group tympanograms were normal, whereas in the remaining 10 ears (12.5%) tympanograms were abnormal. The sensitivity of tympanometry was 94.7% and its specificity was 87.5%. In Table 2, the results for sensitivity, specificity, positive predictive value, and negative predictive value, with their corresponding 95% confidence intervals, are shown.

In 51 ears of the patients (67.1%) otoacoustic emissions were absent. In the remaining 25 ears (32.9%) the mean emission amplitude was reduced, compared to the mean value of the control group (Table 1). In 68 of the 80 ears of controls clear TEOAEs were recorded. Comparison of signal-to-noise ratios by independent sample *t*-test between the two groups showed statistically significant differences. In all cases the values of the patients were lower than the mean value of the controls. In this comparison only the ears with present emissions were included from both groups. The sensitivity of TEOAEs was 67.1% and its specificity was 85% (Table 2). Combination of TEOAEs and tympanometry yielded sensitivity 98.6% and specificity 92.5%, improving further diagnostic accuracy (Table 2). The screening test efficiency values (overall number of true positives and true negatives divided by the total number of ears tested) for TEOAEs, tympanometry, and their combination were, respectively, 76.2%, 91%, and 95.5%.

## 4. Discussion

TEOAEs are a valuable screening tool for hearing impairment, although neither information about the degree or configuration of hearing loss is provided, nor is differential diagnosis between sensorineural and conductive hearing loss possible [3]. TEOAEs are transmitted from the cochlea through the ossicles and tympanic membrane and measured in the external ear canal. Therefore, any middle-ear or outer-ear disorder can practically interfere with transiently evoked otoacoustic emission transmission [5]. It has been reported that artificial manipulation of the middle-ear compliance causes a decrease in the response levels of the otoacoustic emissions [9]. Glattke et al. [10] found that a type B tympanogram precluded recording of transiently evoked otoacoustic emissions. Even the presence of negative tympanometric peak pressure has been found to lower the level of TEOAEs, by approximately 4 dB across 1000 to 4000 Hz [11]. Also, Owens et al. [12] reported that in ears with type B and C tympanograms transiently evoked otoacoustic emissions were absent or had reduced amplitudes and concluded that, in general, the use of TEOAEs is contraindicated in the presence of any middle-ear disorder. Furthermore, Choi et al. [13] reported that middle-ear effusion or a type B of tympanogram impeded measurement of transiently evoked otoacoustic emissions, whereas type A of tympanogram was found to be associated with present emissions. However, Hall III et al. [14] proposed that otoacoustic emission testing should be performed even in the presence of middle-ear disease. Koike and Wetmore [15] found that the status of the middle ear greatly affected transiently evoked otoacoustic emission measures, which was most significant with flat tympanograms, mainly indicative of reduced tympanic membrane mobility and the presence of middle-ear effusion. These authors too encouraged the routine use of transiently evoked otoacoustic emission testing.

In several reports, high failure rates of TEOAEs in cases with flat tympanograms were found. Ho et al. [16] reported failure rates approaching 71-72% in ears with middle-ear effusion and abnormal tympanograms. These authors found good agreement between tympanometry failure and transiently evoked otoacoustic emission failure for the 3- to 5-year-old group of children. Similar rates were found in another report of transiently evoked otoacoustic emission measurements [17], and our rate of approximately 67% failure is in accordance with them. In most of the previously mentioned reports the Liden/Jerger tympanogram classification system was used. Fortunately, quantitive analysis is now possible and objective criteria may be used [6], as in the present study. Dragičević et al. [18] have also used TEOAEs as a hearing screening tool in children with OME, before and after surgery. According to these authors, preoperative TEOAEs were absent in 93.5% of the ears, but were significantly improved postoperatively.

When applying tympanometry and TEOAEs in hearing screening, the sensitivity and specificity of both tests should be considered. The problem in such studies is the absence of a gold standard, as would be the findings of myringotomy in the examined ears. However, this is not possible in most cases, and several authors have used tympanometry as the gold standard, due to its high sensitivity to middle-ear disorders. Taylor and Brooks [19] obtained by this method 60% sensitivity and 91% specificity of TEOAEs compared to tympanometry. Recently, Śliwa et al. [20] combined three diagnostic methods, automated 4-frequency audiometry, TOEAEs and tympanometry, in hearing screening of school children. The authors used conventional tone audiometry as the reference and found high specificity and low sensitivity values for all the tests, but combination of tympanometry and TEOAEs yielded 60% sensitivity and 94% specificity, whereas addition of automated 4-frequency audiometry further improved sensitivity to 70%.

In our study, we used the combination of positive history, positive otoscopic appearance by pneumatic otoscopy, abnormal tympanographic findings, and elevated threshold in pure-tone audiometry as a gold standard to establish diagnosis. According to these, the sensitivity of tympanometry was as high as 96% and its specificity was 85%. When calculating the sensitivity of TEOAEs according to the “pass” criterion, a rate of 67% was obtained, whereas specificity approached 85%. However, in the remaining ears which obtained the “pass” criterion, transiently evoked otoacoustic emission measurements were lower than controls, at a statistically significant level. It appears, thus, that the sensitivity of TEOAEs in otitis media with effusion is high, whenever absolute values of TEAOE measurements are considered and not only a “pass” criterion. Specificity is lower, because TEOAEs are also influenced by inner-ear disease, although this is not quite common in the age group of our study.

Finally, a comment on pure-tone thresholds in standard audiometry should be made. In several reports, mean pure-tone thresholds between 15 and 20 dB HL have been found [21]. In our study mean thresholds were approximately 20–25 dB HL in the standard frequencies of 0.5, 1, 2 and 3 kHz. It appears, thus, that an audiometric criterion of 20 dB would be appropriate to separate normal ears from ears with absent or reduced transiently evoked otoacoustic emissions.

## 5. Conclusion

From this study, it may be concluded that TEOAEs should be included in the diagnostic workup of otitis media with effusion. Their sensitivity in diagnosing middle-ear disease is high when quantitive measures are used, whereas their specificity is lower, because abnormal results may also be found in cases with inner-ear disease. For this reason, TEOAEs should always be used along with tympanometry, because a more meaningful interpretation of transiently evoked otoacoustic emission measures in conjunction with tympanometry results is possible.

## Figures and Tables

**Table 1:** Means and levels of statistical significance (*P*) of pure-tone thresholds and signal-to-noise ratios of transiently evoked otoacoustic emissions (TEOAEs), comparing the ears of patients and the ears of controls.

Frequencies (kHz)	Pure-tone thresholds			Signal-to-noise ratios (TEOAEs)		
	Patient ears	Control ears		Patient ears	Control ear
	(*N* = 72)	(*N* = 80)	*P*	(*N* = 25)	(*N* = 68)	*P*
0.25	30.4	12.5	<0.001	nm*	nm	nm
0.5	28.7	9.4	<0.001	nm	nm	nm
1.0	23.3	8.8	<0.001	5.8	4.6	<0.01
1.5	nm	nm	nm	11.2	6.3	<0.001
2.0	24.7	12.0	<0.001	17.4	7.4	<0.001
3.0	19.8	14.3	<0.01	16.1	8.3	<0.001
4.0	17.5	15.3	ns^†^	16.8	9.0	<0.001
8.0	13.4	14.3	ns	nm	nm	nm

* not measured; ^†^non significant.

**Table 2:** Estimates for the sensitivity, specificity, positive predictive value, and negative predictive value of transiently evoked otoacoustic emissions (TEOAEs), tympanometry, and the combined use of both tests. Numbers in parentheses provide estimates of the 95% confidence intervals.

Statistical measures	TEOAEs	Tympanometry	TEOAEs and tympanometry
Sensitivity (%)	67.1 (55.2–77.1)	94.7 (86.3–98.3)	98.6 (91.8–99.9)
Specificity (%)	85.0 (74.8–91.6)	87.5 (77.7–93.5)	92.5 (83.8–96.9)
Positive predictive value (%)	80.9 (68.7–89.3)	87.8 (78.2–93.6)	92.5 (83.9–96.9)
Negative predictive value (%)	73.1 (62.7–81.5)	94.5 (86.0–98.2)	98.6 (91.7–99.9)
